# Diagnosing necrotizing external otitis on CT and MRI: assessment of pattern of extension

**DOI:** 10.1007/s00405-021-06809-2

**Published:** 2021-04-25

**Authors:** Wilhelmina L. van der Meer, Jérôme J. Waterval, Henricus P. M. Kunst, Cristina Mitea, Sjoert A. H. Pegge, Alida A. Postma

**Affiliations:** 1 Department of Radiology and Nuclear Medicine, Maastricht University Medical Center, P. Debyelaan 25, 6229 HX Maastricht, The Netherlands; 2 Department of Otorhinolaryngology and Head and Neck Surgery, Maastricht University Medical Center, P. Debyelaan 25, 6229 HX Maastricht, The Netherlands; 3 Department of Otorhinolaryngology and Head and Neck Surgery, Radboud University Medical Center, Radboud Institute for Health Sciences, Nijmegen, The Netherlands; 4 GROW-School for Oncology and Developmental Biology, Maastricht University, Maastricht, The Netherlands; 5 Department of Radiology and Nuclear Medicine, Radboud University Medical Center, P. Debyelaan 25, 6229 HX Maastricht, The Netherlands; 6 School for Mental Health & Neuroscience, Maastricht University, Maastricht, The Netherlands

**Keywords:** Necrotizing external otitis, Skull base osteomyelitis, Malignant external otitis, Spreading patterns, CT, MR

## Abstract

**Background and purpose:**

Necrotizing external otitis (NEO) is a serious complication of external otitis. NEO can be classified according to—anterior, medial, posterior, intracranial, and contralateral—extension patterns. Currently there is no consensus on the optimal imaging modality for the identification of disease extension. This study compares NEO extension patterns on MR and CT to evaluate diagnostic comparability.

**Methods:**

Patients who received a CT and MR within a 3-month interval were retrospectively examined. Involvement of subsites and subsequent spreading patterns were assessed on both modalities by a radiologist in training and by a senior head and neck radiologist. The prevalence of extension patterns on CT and MR were calculated and compared.

**Results:**

All 21 included NEO cases showed an anterior extension pattern on CT and MR. Contrary to MR, medial extension was not recognized on CT in two out of six patients, and intracranial extension in five out of eight patients. The posterior extension pattern was not recognized on MR. Overall, single anterior extension pattern (62%) is more prevalent than multiple extension patterns (38%).

**Conclusion:**

All anterior NEO extension pattern were identified on CT as well as MR. However, the medial and intracranial spreading patterns as seen on MR could only be identified on CT in a small number of patients. The posterior spreading pattern can be overlooked on MR. Thus, CT and MR are complimentary for the initial diagnosis and work-up of NEO as to correctly delineate disease extent through the skull base.

## Introduction

Necrotizing external otitis (NEO) is a rare complication of an external auditory canal infection, in which pathogens spread via the fissures of Santorini and/or the foramen of Huschke to the skull base [1, 2]. NEO presents as a therapy-resistant external otitis and is often accompanied by an epithelial defect with partially uncovered bone or granulation tissue in the external auditory canal. Patients at risk for the development of NEO are mainly elderly diabetic patients or the immunocompromised, e.g., patients after organ transplantation. Mortality rates up to 15% have been described in this frail patient population [3]. The spread of NEO through the skull base can affect a wide range of anatomical structures, possibly causing cranial nerve palsies in severely affected cases.

Imaging findings described in relation to NEO are diverse and can involve fat infiltration, soft tissue swelling, venous (sinus) thrombosis and/or osseous destruction of the skull base. The afflicted soft tissues and osseous structures can be classified on CT and MR according to—anterior, medial, posterior, intracranial, and contralateral—spreading patterns [4–7] (Table 1). The anterior spreading pattern, with involvement of the retrocondylar fat, is most commonly identified in NEO [4, 6].

**Table 1:** NEO extension patterns and associated subsites for soft tissue and bone tissue [7]

Spreading pattern	Soft tissue	Bone and Joint tissue
Anterior	Retrocondylar fat Subtemporal fat Masticator space Parotid gland Facial nerve	Tympanic and squamous part of temporal bone Temporomandibular joint Stylomastoid foramen
Medial	Parapharyngeal fat Nasopharyngeal thickening Preclival soft tissue	Sphenoid bone Clivus Petrous apex Foramen lacerum Jugular foramen
Posterior	–	Mastoid part of the temporal bone
Intracranial	Sigmoid sinus Jugular vein Intracranial carotid artery Dural enhancement	Intracranial surface of the petrous and mastoid part of the temporal bone Jugular fossa Petroclival synchondrosis
Contralateral	Soft tissue involvement past midline	Bone and joint involvement past midline

The majority of otorhinolaryngologists diagnose NEO on a combination of clinical symptoms, examination results of the external auditory canal, and imaging results [8]. At the moment, there is no gold standard for NEO diagnosis or work-up. The optimal diagnostic imaging strategy remains subject to discussion as reflected by several surveys [8, 9]. However, the majority of survey respondents indicated CT as the first preferred imaging modality for the establishment of NEO diagnosis, and CT and/or MR for the assessment of disease extent [9].

A developing role for the evaluation of disease extent may be found in nuclear medicine [10]. Nuclear medicine is known for imaging functional abnormalities, such as inflammation, rather than just anatomical disease extent. However, functional assessment can be performed by a range of modalities, such as skeletal scintigraphy with single-photon emission computed tomography (SPECT), FDG—positron emission tomography (PET), PET combined with CT or MRI, and even leucocyte labelling. At the moment, nuclear imaging is mainly used for monitoring therapy effect, but the optimal nuclear diagnostic method for the evaluation of NEO disease extent and follow-up is unclear [11].

Currently, the role of CT or MR for the correct delineation of NEO extension remains unclear. The goal of this study is to investigate the relative strengths and weakness for CT and MR per extension pattern, thus aiding the clinician to choose a correct imaging method for the optimal recognition of NEO.

## Method

A retrospective PACS survey was conducted with key words “necrotizing external otitis,” “malignant external otitis,” and “skull base osteomyelitis” on CT and MR from 2011 to 2019. All radiologic identified NEO cases were prior inclusion confirmed by an otorhinolaryngologist. NEO patients were included when a (HR)CT of the temporal bone and a supplementary MR was present. Patients were excluded from the study if (HR)CT and MR studies were performed with an interval difference of more than 3 months. Patient records were reviewed for diseases associated with NEO, clinical complaints, and ear canal biopsy results.

All non-contrast enhanced CT scans were reviewed with axial and coronal bone reconstruction with a slice thickness of 0.4–1 mm. When present, CT imaging with soft tissue reconstructions were reviewed. MR 1.5 T scans consisted of T1, T1-gado, T2, DWI/ADC sequences. CT scans and MR of the temporal bone were assessed on NEO spreading patterns and known subsites as described in literature (Table 1). All subsites were assessed for possible disease involvement by a dedicated head and neck radiologist in training and by a senior head and neck radiologist with over 10 years of experience. Subsites were regard as truly involved when both reviewers had independently scored the structure as abnormal. Frequency and percentages of involved subsites were noted. Diagnostic agreement between CT and MR was analyzed with Cohen’s kappa, with values between 0.4 and 0.6 regarded as moderate, 0.6–0.8 as substantial, and 0.8–1.0 as almost perfect agreement.

## Results

CT was the initial exam in all cases. In total 21 patients received an MR study within a 3 months interval of the CT. The imaging studies included 17 male and 4 female patients with a mean age of 76.4 years (SD 11.0 years). In total 14 of 21 patients were at increased risk for NEO due to diabetes or immunodeficiency. *Pseudomonas aeruginosa* was the most common cultured pathogen (57%). The difference between CT and MR studies ranged from 0 to 86 days, with a mean time difference of 18.7 days (SD 22.8 days) (Table 2).

**Table 2:** Patient characteristics and NEO extension patterns as diagnosed on CT and MR

Patient	Sex	Age (years)	Anterior		Medial		Posterior		Intracranial		Contralateral		Imaging time difference (days)
			CT	MR	CT	MR	CT	MR	CT	MR	CT	MR	
1	M	67	+	+	+	+	+	−	+	+	+	+	6
2	M	70	+	+	−	−	−	−	−	−	−	−	0
3	M	65	+	+	+	+	−	−	−	+	−	−	18
4	M	86	+	+	+	+	−	−	−	+	−	−	55
5	F	79	+	+	−	−	−	−	−	−	−	−	86
6	M	64	+	+	+	+	−	−	−	−	−	−	9
7	F	90	+	+	−	−	−	−	−	−	−	−	9
8	M	46	+	+	−	−	+	−	+	−	−	−	1
9	M	85	+	+	+	−	−	−	+	−	−	−	7
10	M	85	+	+	+	+	−	−	−	−	−	−	10
11	F	81	+	+	+	−	−	−	−	−	−	−	7
12	M	83	+	+	−	−	−	−	−	−	−	−	17
13	M	83	+	+	−	+	−	−	−	+	−	−	8
14	M	79	+	+	−	−	−	−	−	−	−	−	15
15	M	78	+	+	−	−	−	−	−	−	−	−	5
16	M	84	+	+	−	−	−	−	−	−	−	−	8
17	M	65	+	+	−	−	−	−	−	−	−	−	6
18	M	91	+	+	−	−	−	−	−	−	−	−	63
19	F	66	+	+	−	-	−	−	−	+	−	−	18
20	M	82	+	+	−	+	−	−	−	+	−	−	48
21	M	76	+	+	−	−	−	−	−	−	−	−	9
Overall (*N*)			21	21	7	7	2	0	3	6	1	1	

+ Positive finding, − negative finding

### CT

All 21 NEO patients showed an anterior extension pattern on CT. The anterior extension pattern was solely present in 13/21 (62%) cases, and combined with other extension patterns in 8/21 (38%) cases (Table 2). Retrocondylar, subtemporal, and masticator space fat infiltration were the most affected subsites of the anterior extension pattern on CT (Table 3). The most commonly affected subsites of the medial extension pattern were parapharyngeal fat planes, the jugular foramen, and the preclival fat. The posterior spreading pattern solely consists of one subsite; cortical destruction of the mastoid part of the temporal bone which was found in two cases. The most affected subsite of the intracranial spreading pattern was the jugular fossa. No changes were observed at the petroclival synchondrosis.

**Table 3:** Observed NEO extension patterns and subsites for CT and MR

NEO spreading pattern	CT (21) *n* (%)	MR (21) *n* (%)
Anterior	21 (100)	21 (100)
Retrocondylar fat	19 (90)	18 (86)
Subtemporal fat	13 (62)	16 (76)
Masticator space	10 (48)	12 (57)
Temporomandibular joint	9 (43)	7 (33)
Stylomastoid foramen (CN VII)	8 (38)	11 (52)
Parotid gland	7 (33)	6 (29)
Temporal fossa	4 (19)	1 (5)
Medial	7 (33)	7 (33)
Parapharyngeal fat	5 (24)	6 (29)
Jugular foramen (CN. IX, X, XI)	4 (19)	4 (19)
Preclival fat	3 (14)	6 (29)
Clivus	2 (10)	5 (24)
Sphenoid	1 (5)	2 (10)
Petrous apex	1 (5)	6 (29)
Foramen lacerum	1 (5)	6 (29)
Posterior	2 (10)	0 (0)
Mastoid process of temporal bone	2 (10)	0 (0)
Intracranial	3 (14)	6 (29)
Jugular fossa	3 (14)	2 (10)
Petroclival synchondrosis	0 (0)	3 (14)
Dural enhancement	–^a^	0 (0)
Patent veins	–^a^	3 (14)
Contralateral	1 (5)	1 (5)

^a^Not assessable on CT

### MR

The anterior spreading pattern on MR was also present in all 21 NEO patients. A sole anterior extension pattern was found in 62% (13/21) of the cases, and in combination with additional spreading patterns in 8/21 (38%) cases (Table 2). The most commonly affected subsites of the anterior spreading pattern on MR were retrocondylar, subtemporal, masticator space fat infiltration, the stylomastoid foramen (Table 3). The most common affected subsites for the medial spreading pattern were the parapharyngeal and preclival fat, petrous apex, and foramen lacerum. None of the patients with a posterior spreading pattern could be identified on MR imaging. The most frequently affected site of the intracranial spreading pattern was the petroclival synchondrosis. None of the NEO cases in this study showed dural enhancement.

### Spreading pattern comparison

All 21 anterior NEO extension cases were recognized on both CT and MR. The posterior extension pattern could only be identified on CT. The medial and intracranial extension cases could be better identified on MR. The medial spreading pattern showed a Cohen’s kappa agreement in 17/21 cases (81%), with a *k* of 0.571. The intracranial spreading pattern showed a Cohen’s kappa agreement of 14/21 (67%) with a *k* of 0.039. The contralateral and posterior patterns were, respectively, present in 1 and 2 cases, respectively, thus Cohen’s kappa agreement could not be adequately determined.

## Discussion

In daily practice (HR)CT of the temporal bone is the common diagnostic modality for patients suspected of NEO. It is essential to know whether CT and MR can accurately identify the presence of NEO and its extension pattern, as for example the intracranial extension correlates with poor clinical outcome [3]. Thus, early identification of extension patterns can possibly help control fatal NEO outcome.

Patients with an anterior NEO spreading pattern could all be identified on CT as well as on MR. No discrepancies were, therefore, found for the anterior spreading pattern when comparing CT and MR imaging in our patient group. One of the early findings of anterior extension is infiltration of the retrocondylar fat pad [6]. The results of our study also show the retrocondylar fat is the most common involved subsite in the anterior spreading pattern on CT as well as MR. However, the anterior spreading pattern cannot solely be based upon retrocondylar fat infiltration as there were patients within this study who showed subtemporal fat infiltration without effacement of retrocondylar fat planes. The effacement of the subtemporal fat planes on (HR)CT has also been described as one of the early signs of NEO, although it is unclear if this was found isolated from retrocondylar fat infiltration [12].

**Fig. 1:**
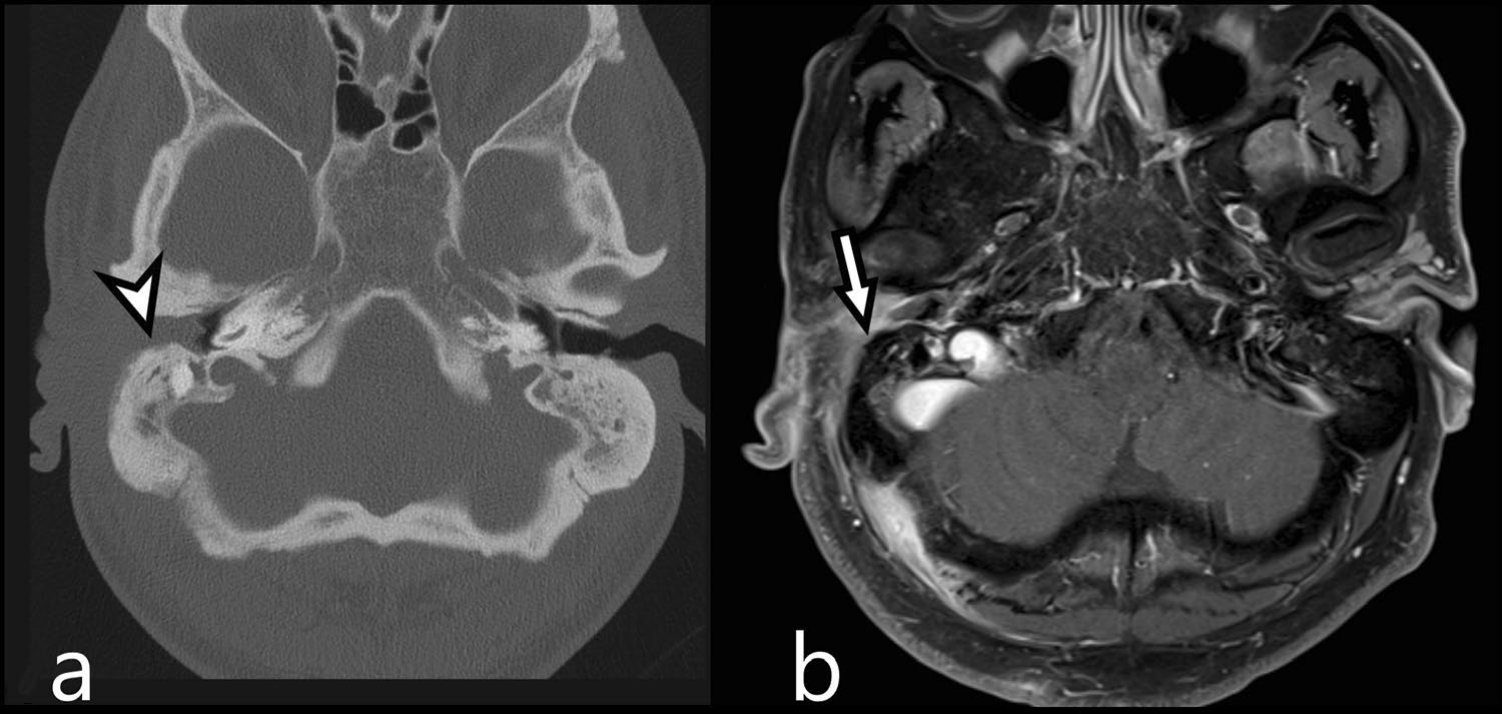
Example of a posterior spreading pattern: a 46-year-old male presented with right sided otalgia, jaw pain and N.VII. paralysis. The CT shows subtle destruction of the mastoid part of the temporal bone (**a**; arrow head). MR T1-fs gado sequences show enhancement of the external ear but no signal abnormalities of the mastoid (**b**; arrow). The increased signal intensity in the venous sinus is due to slow flow

Patients with a posterior spreading patterns were not found by subsequent MR studies, probably as the posterior spreading pattern is solely based upon cortical destruction of the mastoid of the temporal bone, a structure which cannot be optimally evaluated on MR (Fig. 1). In contrast several patients with a medial or an intracranial spreading pattern were not identified on CT but solely on MR. An increased detection of medial extension on MR, in comparison to CT, can be mainly explained by the osseous subsite changes of, for example, the petrous apex and clivus (Table 3). Bone destruction on CT is recognized by cortical irregularities, whereas MRI is sensitive for medullary signal changes. Improved recognition of intracranial subsites can mainly be explained by dural enhancement and venous sinus thrombosis (Table 3). These specific intracranial subsites cannot be optimally evaluated by non-contrast CT imaging, with or without the presence of soft tissue kernels, which warrant the use of MRI. However, a CT with contrast remains recommended when MR contraindications are present.

At the moment the definition and application of NEO spreading patterns remains a matter of debate. Past studies classified the jugular vein as an intracranial structure [1], although anatomically it is located in the neck and not truly intracranial. In contrast, some studies classify the clivus as a midline structure and some as an intracranial structure [4]. Overall the most discrepancies can be found for the intracranial subsites, as one could argue that the jugular foramen, jugular fossa and the petroclival synchondrosis can be better classified as skull base.

**Fig. 2:**
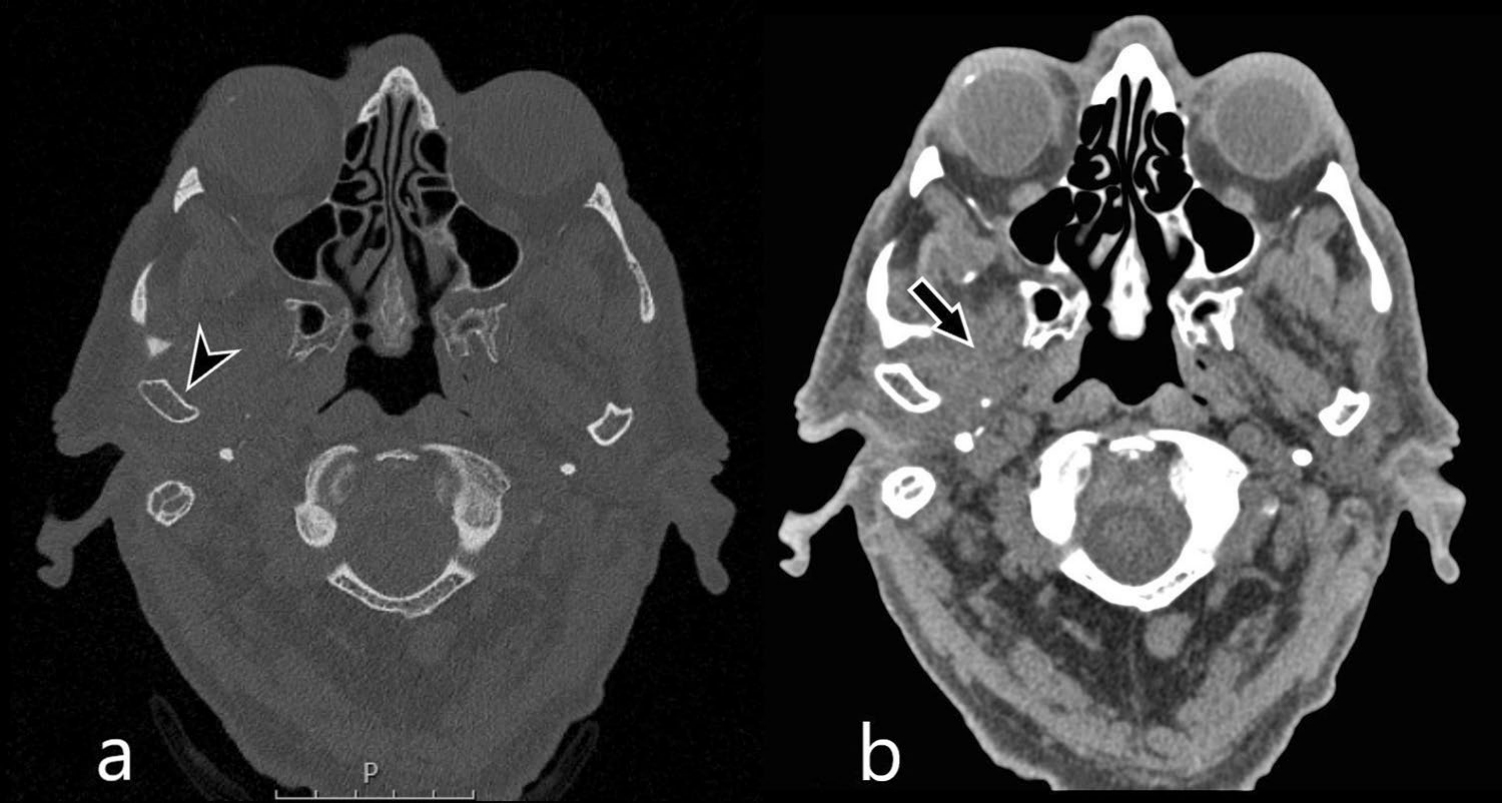
Illustration of the diagnostic value of soft tissue kernels: an 84-year-old male patient with presenting with right sided otalgia and jaw pain. Bone reconstructions show minimal soft tissue swelling and no apparent osseous destruction of the external ear canal and temporomandibular joint (**a**; arrow head). Soft tissue kernel reconstructions show an evident effacement of the masticator space (**b**; arrow). The patient was identified as NEO by the aid of soft tissue kernels

Identification of NEO on CT can be difficult and spreading outside the external ear canal can easily be missed. False negative CT findings have previously been described in literature with rates up to 41% [5]. The improved rate in our cohort can possibly be explained due to diagnostic review by dedicated head neck specialists, and the addition of tissue reconstruction kernels. Typically CT temporal bone studies only use a kernel optimized for the assessment of bone structures. The standard use of soft tissue kernels increases the sensitivity for soft tissue involvement in comparison to conventional CT reconstructions (Fig. 2). A total of 7/21 CT studies (33%) used an additional soft kernel reconstruction, thus possibly increasing the recognition of soft tissues affected by NEO. Alternatively, patients with mild clinical symptoms may not have received diagnostic imaging studies, possibly resulting in a cohort with relative severe and thus apparent imaging abnormalities on CT and MR.

The number of NEO patients in this study, although limited, is consistent with other spreading pattern studies [4, 6]. The inclusion criteria of this study required a CT temporal bone and MR imaging within a time period of 3 months. The inclusion criteria were not met for 24 patients, mostly due to the absence of MR studies, illustrating the underlying problem that there is no consensus on which and when to use imaging modalities. The relatively large time interval between imaging studies may also have influenced the reported spreading patterns. It cannot be excluded that NEO showed improvement or progression during this time period.

Clinically, the patients with an extensive spreading pattern were more affected by cranial nerve palsies (such as paralysis of the facial nerve) often persisting after the treatment. Currently the prognostic value of cranial nerve involvement in terms of mortality is unclear [1]. All patients irrespective of their initial spreading pattern received intravenous beta-lactam antibiotics such as piperacillin/tazobactam or meropenem for a minimal duration of 6–8 weeks. Patients with more extensive spreading patterns showed a need for prolonged antibiotic treatment and thus longer follow-up. At the moment, the duration of antibiotic treatment is mainly determined by the otolaryngologist via clinical symptoms and imaging findings. However, as previously mentioned, an additional role may be found in nuclear medicine for follow-up of NEO disease extent and activity [10, 11]. Hopefully, future research findings may guide clinicians in the optimal decision for treatment duration and follow-up. This study shows that the presence of the anterior NEO spreading route, with the aid of soft tissue kernels, can be correctly determined on CT. However, the presence of spreading routes other than or additional to the anterior spreading route cannot be excluded by the sole use of CT. The sole use of MR imaging, although apt for anterior, medial and intracranial extension patterns can possible miss posterior extension cases. Thus, the optimal imaging modality for NEO diagnosis and work-up remains the complementary use of CT and MR.

## Conclusion

Necrotizing external otitis extension patterns and its respective subsites can be assessed by CT and MR. The anterior spreading pattern can be correctly identified on both CT and MR when carefully evaluating specific subsites with the aid of soft tissue kernels. However, extension and evaluation beyond this pattern requires imaging using both CT and MR. Thus, CT and MR are complimentary for the initial diagnosis and work-up of NEO as to correctly delineate disease extent through the skull base.

## Data Availability

The data that support the findings of this study are available from the corresponding author, upon reasonable request.

## Abbreviations

NEO: Necrotizing external otitis
